# Breast cancer stem cell selectivity of synthetic nanomolar-active salinomycin analogs

**DOI:** 10.1186/s12885-016-2142-3

**Published:** 2016-02-23

**Authors:** Xiaoli Huang, Björn Borgström, Sebastian Kempengren, Lo Persson, Cecilia Hegardt, Daniel Strand, Stina Oredsson

**Affiliations:** Department of Biology, Lund University, Lund, Sweden; Department of Chemistry, Center for Analysis and Synthesis, Lund University, Lund, Sweden; Department of Experimental Medical Science, Lund University, Lund, Sweden; Division of Oncology and Pathology, Department of Clinical Sciences Lund, Lund University Cancer Center/Medicon Village, Lund, Sweden

**Keywords:** Salinomycin, Salinomycin analogs, Breast cancer stem cells, Migration, Mesenchymal to epithelial transition

## Abstract

**Background:**

Cancer stem cells (CSCs) have been invoked in resistance, recurrence and metastasis of cancer. Consequently, curative cancer treatments may be contingent on CSC selective approaches. Of particular interest in this respect is the ionophore salinomycin, a natural product shown to be 100-fold more active against CSCs than clinically used paclitaxel. We have previously reported that synthetic salinomycin derivatives display increased activity against breast cancer cell lines. Herein we specifically investigate the CSC selectivity of the most active member in each class of C20-*O*-acylated analogs as well as a C1-methyl ester analog incapable of charge-neutral metal ion transport.

**Methods:**

JIMT-1 breast cancer cells were treated with three C20-*O*-acylated analogs, the C1-methyl ester of salinomycin, and salinomycin. The effects of treatment on the CSC-related CD44^+^/CD24^−^ and the aldehyde dehydrogenase positive (ALDH^+^) populations were determined using flow cytometry. The survival ability of CSCs after treatment was investigated with a colony formation assay under serum free conditions. The effect of the compounds on cell migration was evaluated using wound-healing and Boyden chamber assays. The expression of vimentin, related to mesenchymal traits and expression of E-cadherin and β-catenin, related to the epithelial traits, were investigated using immunofluorescence microscopy.

**Results:**

Treatment with each of the three C20-acylated analogs efficiently decreased the putative CSC population as reflected by reduction of the CD44^+^/CD24^−^ and ALDH^+^ populations already at a 50 nM concentration. In addition, colony forming efficiency and cell migration were reduced, and the expression of the epithelial markers E-cadherin and β-catenin at the cell surface were increased. In contrast, salinomycin used at the same concentration did not significantly influence the CSC population and the C1-methyl ester was inactive even at a 20 μM concentration.

**Conclusions:**

Synthetic structural analogs of salinomycin, previously shown to exhibit increased activity against cancer cells, also exhibited improved activity against CSCs across several assays even at nanomolar concentrations where salinomycin was found inactive. The methyl ester analog of salinomycin, incapable of charge-neutral metal ion transport, did not show activity in CSC assays, lending experimental support to ionophoric stress as the molecular initiating event for the CSC effects of salinomycin and related structures.

**Electronic supplementary material:**

The online version of this article (doi:10.1186/s12885-016-2142-3) contains supplementary material, which is available to authorized users.

## Background

Breast cancer is the leading cause of cancer death among women worldwide. Often, this outcome is a consequence of recurrence following years of disease-free life after a completed initial treatment [1]. Recurrence has been linked to certain treatment resistant cancer cells, coined cancer stem cells (CSCs), which share many of the properties associated with regular stem cells including self-renewal and differentiation [2]. Several studies have shown enrichment of CSCs following conventional chemotherapeutic treatment, both in vivo and in cancer cell lines [3, 4]. This has lead to the proposal that the chemotherapeutic drugs mainly target bulk cancer cells while sparing cells with CSC properties [5, 6]. Curative treatments may therefore be contingent on therapies that target both CSCs and bulk cancer cells, presumably by a combination of conventional therapies and CSC selective drugs. Of particular interest in a breast cancer context is the natural product salinomycin. Salinomycin was identified in a screen for breast CSC inhibition [7] and has subsequently been shown to inhibit CSCs of many cancer types [8–10]. Salinomycin has been shown to inhibit cell migration and cell proliferation as well as inducing apoptosis and autophagy [11–15]. Proposed mechanisms include inhibition of Wnt [16–18] and Hedgehog signaling [19], inhibition of multidrug efflux systems [20, 21], induction of reactive oxygen species [22, 23], cleavage of poly-ADP-ribose polymerase [24, 25], and induction of DNA damage [26]. However, the actual molecular initiating event in the CSC or cancer cell adverse outcome pathways has not been clarified. As evidenced by the number of different mechanisms proposed, the actual initiating event may be obfuscated by the high treatment concentrations often used. It is well known that salinomycin is a potent ionophore with the capacity to transport alkali metal ions and it has been suggested that it acts in biological membranes by promoting potassium ion efflux [27, 28].

Despite the mechanistic uncertainty, salinomycin has been used in limited clinical trials showing positive responses [28, 29]. Towards increasing the clinical relevance of salinomycin, there is thus considerable interest in more active and selective structures acting through the same mechanism as well as developing an understanding of how such compounds selectively reduce CSC populations. We have previously demonstrated that selective chemical modification of salinomycin at the C20 hydroxyl group can be used to access significantly more active analog structures with IC_50_ values down to below one fifth of that of the native structure in two breast cancer cell lines [30]. In fact, these compounds represent the most active salinomycins known. In addition, chemical modifications at other positions of salinomycin as well as the anti proliferative effects of such derivatives have been described [30–35].

**Fig. 1:**
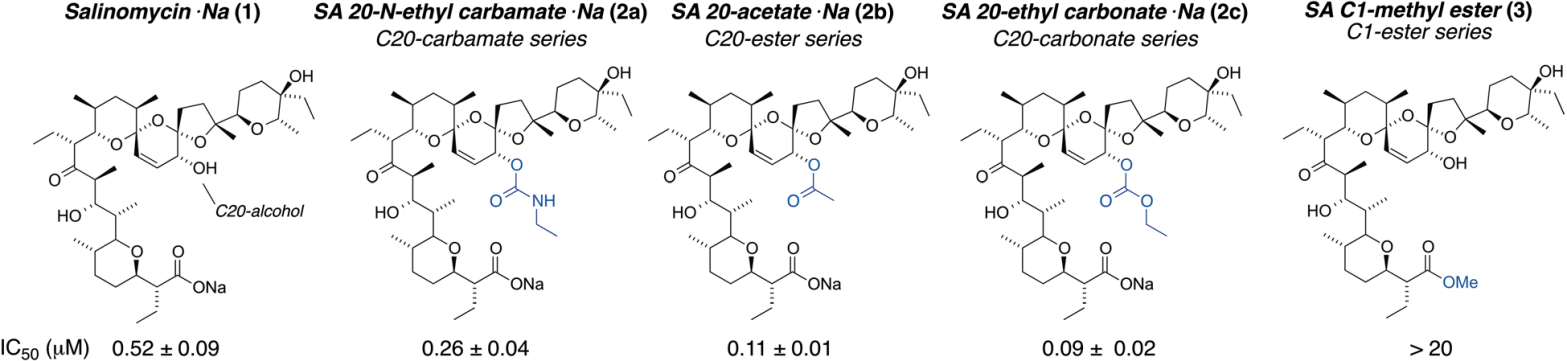
Chemical structures of salinomycin (SA) and synthetic salinomycin analogs. Structural differences compared to salinomycin are highlighted in blue. IC_50_ in JIMT-1 cells given as mean ± SEM [30]

Herein, we show that the enhanced activity of the most active analog in each of the ester, carbamate and carbonate series of C20-*O*-acylated structures identified in our previous study (Fig. 1) also translates to superior selectivity against putative CSCs as compared to salinomycin itself. Treatment with each of the C20-acylated analogs efficiently reduced traits related to CSC activity in three different assays already at low nanomolar concentrations where salinomycin itself did not show activity. The unique potency of C20-*O*-acylated structures should be of interest in clinical settings where specific pharmacological activity at low concentrations is highly favorable. Of mechanistic significance, salinomycin C1-methyl ester (Fig. 1), a structure lacking the primary ion-binding motif of salinomycin, the carboxylic acid moiety, was also evaluated in parallel. Despite sharing the basic molecular framework of salinomycin, this structure, which is incapable of charge-neutral metal ion transport, did not show CSC selective properties. This finding is in line with disturbances in ion gradients as the main molecular initiating event and suggests that CSCs can, at least in part, be inhibited by such a mechanism.

## Methods

In this study, the well-established commercially available human breast cancer cell line JIMT-1 (ACC589) was used and no ethical approval was required.

### Cell line and culturing conditions

The human breast carcinoma cell line JIMT-1 (ACC589) was purchased from the German Collection of Microorganisms and Cell Cultures (DSMZ) and was routinely cultured in Dulbecco’s modified Eagle’s medium/Ham’s F-12 nutrient mixture (1/1) supplemented with 10 % fetal calf serum (FCS), nonessential amino acids (1 mM), insulin (10 μg/ml), penicillin (100 U/ml) and streptomycin (100 μg/ml). The JIMT-1 cell line was established from a pleural metastasis of a 62-year old patient with breast cancer who was clinically resistant to trastuzumab [36]. It is a well-characterized estrogen receptor negative and HER2 amplified cell line belonging to the HER2 plus sub-type of breast cancer [36, 37]. The JIMT-1 cell line contains phenotypically different cell populations based on expression of CSC markers CD44, CD24 and ALDH [38]. This cell line has been used in several studies to investigate effects on the CSC subpopulation [39–42].

The JIMT-1 cell cultures were kept at 37 °C in a humidified incubator with 5 % CO_2_ in air. If not specified below, the cells were seeded at a density of 20,000 cells/cm^2^ using 0.2 - 0.3 ml of medium per cm^2^ in a tissue culture dish with appropriate size for the respective assays. Active compounds were added 24 h after seeding, and the cells were sampled for the various analyses after 72 h of treatment. Cell counting was performed in a hemocytometer.

### Compounds

Technical grade salinomycin (12 %) was obtained from Chemtronica AB. This material was purified as described previously [30] and salinomycin was isolated and used as its sodium salt. Salinomycin analogs **2a**-**c** and methyl ester **3** were synthesized according to [30]. Analogs **2a**-**c** were used as the respective sodium salt. The compounds were diluted in 100 % dimethyl sulfoxide (DMSO) to a 10 mM stock solution which was kept at 4 °C. The compounds were diluted in phosphate-buffered saline (PBS) to give working solutions at appropriate concentrations. The controls were supplemented with PBS containing DMSO at the same concentrations as in the working solutions of the compounds. The final DMSO concentration was 0.2 % when using the C20-*O*-acylated analogs at the IC_50_ concentration [30]. An IC_50_ was not obtained for the salinomycin C1-methyl ester [30] and it was used at a 20 μM concentration in experiments comparing effects at IC_50_. The DMSO concentration was 0.0005 % when using the compounds at a 50 nM concentration.

### Cell cycle analysis by flow cytometry

After 72 h of treatment, cells were harvested by trypsinization and fixed in ice-cold 70 % ethanol for at least 1 h at −20 °C. The cells were then stained and analyzed as described previously [43]. The assay does not distinguish between G_0_ and G_1_ cells, thus, when G_1_ is mentioned in the text and figures it denotes both populations. Since the method removes the cell membranes, cells in mitosis (M) are not included in the assay.

### Western blot

After 72 h of treatment, cells were harvested using Accutase (Sigma) for 10 min at 37 °C, then counted, pelleted and stored at −80 °C until further use. The Western blot was performed as described previously [42].

### Cell surface markers identified by flow cytometry

Cells were harvested using Accutase and identified based on their expression of the cell surface markers CD44 and CD24 using a BD Accuri C6 as described previously [35]. Antibodies used for flow cytometry (CD44-fluorescein isothiocyanate (FITC)-conjugated (clone G44-26), CD24-phycoerythrin (PE)-conjugated (clone ML5) and PE-conjugated or FITC-conjugated mouse IgG1 isotype controls (MOPC-21)) were purchased from BD Biosciences. Labeling with 7-aminoactinomycin D (7AAD) was initially used to gate only live cells, but this staining could be omitted since dead cells were typically not seen and usage of 7AAD should be minimized as this is a potent carcinogen. In addition, the cell harvesting procedure included a rinsing step that removed detached dead cells.

### ALDEFLUOR assay

The ALDEFLUOR kit (Stem Cell Technologies) was used according to the manufacturer’s protocol. The cells were harvested using Accutase and after cell counting, two test tubes containing 200,000 cells in assay buffer were prepared for each sample. One of the test tubes was used as negative control receiving the specific ALDH inhibitor diethylaminobenzaldehyde (DEAB). Then the ALDH substrate BODIPY-amino acetaldehyde was added to both tubes which were incubated for 45 min at 37 °C. After incubation, the cells were pelleted by centrifugation and the cells were resuspended in 500 μl assay buffer before analysis in a BD Accuri C6 flow cytometer. DEAB-treated cells served as control to set the ALDH^+^ region for each sample. The CFlow software was used to evaluate the data.

### Colony formation assay in soft agar

The colony formation assay was performed as described previously [35].

### Wound-healing assay

JIMT-1 cells were seeded in 6-well plates (125,000 cells/cm^2^) and allowed to attach for 24 h resulting in a confluent layer of cells. The medium was removed and three scratches (wound areas) were made in the cell layer with a sterile 200 μl pipette tip. After washing twice with PBS, medium without FCS containing 0.0005 % DMSO (control) or the respective compound at a concentration of 50 nM was added to the wells. The scratch area was photographed directly (time 0), as well as after 24, 48 and 72 h of treatment in an inverted phase contrast microscope. The migration was estimated by measuring the scratch area at 0, 24, 48 and 72 h of treatment with ImageJ 1.47v software. The scratch area at each time point was divided with the area at 0 h to obtain a measure of wound closure. The wound area was defined as 0 % closed at 0 h for each sample.

### Boyden chamber cell migration assay

JIMT-1 cells were detached with Accutase, the cell number determined by counting in a hemocytometer and the cells were then diluted in serum free medium to a concentration of 50,000 cells/250 μl. The cell suspension was incubated for 30 min at 37 °C in a water bath to acclimatize the cells to the serum free medium. The cells in serum-free medium were then seeded (50,000 cells) in tissue culture inserts with membranes having 8.0 μm pores (BD Falcon^TM^ Cell Culture Inserts). The inserts were then placed into the wells of 24-well plates containing 500 μl medium with 10 % FCS. The compounds were added to both the inserts and to the wells to a concentration of 50 nM while control received 0.0005 % DMSO. The 24-well plates were incubated in the CO_2_ incubator for 24 h. The cells that had migrated through the pores of the membrane were fixed and stained with cell stain (Millipore, part No. 90144) for 20 min. The trans wells were rinsed in Millipore water and non-migrated cells removed with cotton swabs. The membranes were then left to air-dry. Ten photos were taken of each membrane in an inverted phase contrast microscope. Migrated cells were counted and the mean of migrated cells for each sample was compared to control.

### Immunofluorescence microscopy

JIMT-1 cells were plated on poly-L-lysine-coated glass slides and treated with 50 nM compound or 0.0005 % DMSO as control for 72 h. After fixation in 3.7 % paraformaldehyde (in PBS) for 15 min and subsequent washing in PBS, the cells were permeabilized with PBS containing 1 % Tween 20 and 1 % bovine serum albumin in a single step. The cells were incubated with primary antibody against vimentin (1:100), β-catenin (1:500) or E-cadherin (1:100) for 1 h at room temperature. Antibodies against vimentin (ab8978) and E-cadherin (ab1416) were purchased from Abcam. Antibody against β-catenin (610154) was purchased from BD Biosciences. After washing, the cells were incubated for 1 h with the Alexa Fluor 488 goat anti mouse (1:300) antibody (Invitrogen). Slides were counter-stained with bisbenzimide (Hoechst 33258) (1 μg/ml in PBS) for 2 min and finally washed with PBS before mounting. The cells were viewed in an Olympus/Nikon epifluorescence microscope (Olympus Optical Co. Ltd.) and photos were taken with a digital camera (Nikon Imaging Japan Inc.). Each slide was photographed at randomly chosen areas (at least 8 areas).

### Statistical analysis

The software program GraphPad Prism 6 was used for statistical analysis. A one-way ANOVA test, using the no matching or paring option, was used to detect difference between control and treated samples. To compare the mean of each column with the mean of a control, the Dunnet multiple comparisons test was applied using a 95 % confidence interval.

## Results

### C20-*O*-acylated analogs are more efficient than salinomycin against breast CSCs at a 50 nM concentration

**Fig. 2:**
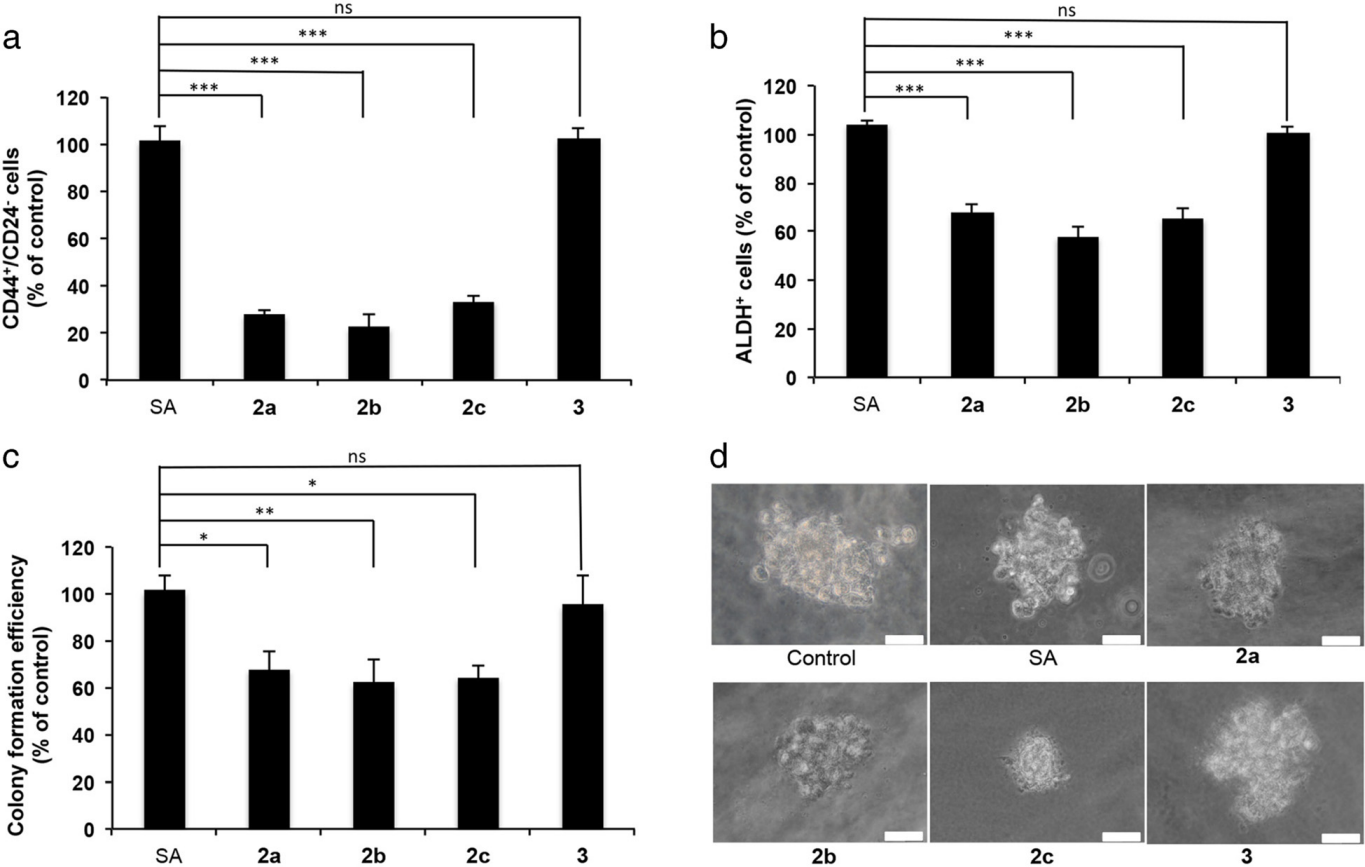
The analogs **2a**–**c** are more efficient than salinomycin against CSCs at a 50 nM concentration. **a** The CD44^+^/CD24^−^ population evaluated using flow cytometry after 72 h of treatment. **b** The ALDH^+^ population evaluated using flow cytometry after 72 h of treatment. **c** Colony forming efficiency evaluated using a serum free soft agar assay. The cells were treated for 72 h and then reseeded at cloning density. The colonies were counted after 2 weeks of incubation. **d** Colonies obtained from the serum free soft agar assay. Bars = 40 μm. Data are represented as mean ± SEM for *n* = 4. *P < 0.05, **P < 0.01, ***P < 0.001. ns: no significant difference. SA: salinomycin, **2a**: carbamate, **2b**: acetate, **2c**: carbonate and **3**: C1-methyl ester

Breast CSCs have been identified based on a high expression of CD44 paired with absent/low expression of CD24 on the cell surface [44]. We have previously shown that salinomycin (**1**) gave the highest selective activity against CD44^+^/CD24^−^ cells at ~ IC_25_ in the breast cancer cell line JIMT-1 [35]. Thus, we decided to investigate the CSC activity of the analogs **2a**-**c** in this cell line using a 50 nM concentration, which corresponds to the approximate IC_25_ values for these structures. At this concentration, we found that treatment with each of the C20 analogs efficiently reduced the CD44^+^/CD24^−^ subpopulation while treatment with salinomycin or the salinomycin C1-methyl ester **3** had no observable effect (Fig. 2a and Additional file 1: Figure S1). We have previously shown that treatment with varying concentrations of salinomycin gives a U-shaped dose response curve with the maximum reduction of the CD44^+^/CD24^−^ around IC_25_ [35], and this was also found to be the case for the more active analogs as exemplified by carbamate **2a** (Additional file 2: Figure S2). To account for this effect in JIMT-1 cells following treatment with salinomycin or either of the two related structures SY-1 and 18,19-dihydro SY-1, we have previously discussed the relation between a decrease in the total cell number and the decrease in the population of CD44^+^/CD24^−^ cells at various concentrations [35]. At concentrations up to IC_25_, the reduction in cell number only originated from a selective reduction of CD44^+^/CD24^−^ cells but at concentrations above IC_25_, all populations were affected and at higher concentrations there was little or no reduction in the CD44^+^/CD24^−^ population resulting in a U-shape dose response curve [35].

The most common trait of CSCs of different tumor origins appears to be expression of aldehyde dehydrogenase (ALDH) [45]. Treatment with 50 nM of C20 analogs efficiently reduced the ALDH^+^ population while treatment with salinomycin or the salinomycin C1-methyl ester had no observable effect (Fig. 2b and Additional file 3: Figure S3). In contrast to the U-shaped dose response curve for CD44^+^/CD24^−^, varying the concentrations of salinomycin gave a dose dependent reduction in ALDH^+^ cells (Additional file 4: Figure S4).

Colony forming efficiency in serum free soft agar is a functional assay which has been used to investigate survival potential of cells with stem cell properties in cancer cell lines [46]. We found that treatment with analogs at a 50 nM concentration reduced the colony forming efficiency and colony size of JIMT-1 cells while treatment with salinomycin or the C1-methyl ester again had no observable effect at the same concentration (Fig. 2c and d).

### Treatment with C20 analogs increase the expression of epithelial markers and decrease the expression of vimentin

CSCs have been shown to exhibit properties that arise in the epithelial to mesenchymal transition (EMT) process and these properties contribute to the metastasis of cancer [47–49]. Compounds that reduce the mesenchymal properties of CSCs should be of interest as a strategy for preventing metastasis. Some important traits of EMT are the loss of E-cadherin and β-catenin on the cell surface. β-Catenin is localized at cell-cell junctions and its association with E-cadherin leads to a stable epithelial structure. A loss of β-catenin from the cell membrane borders is seen in migratory mesenchymal cells [50, 51]. An additional marker for cell mobility is an increased expression of the mesenchymal cytoskeletal protein vimentin.

**Fig. 3:**
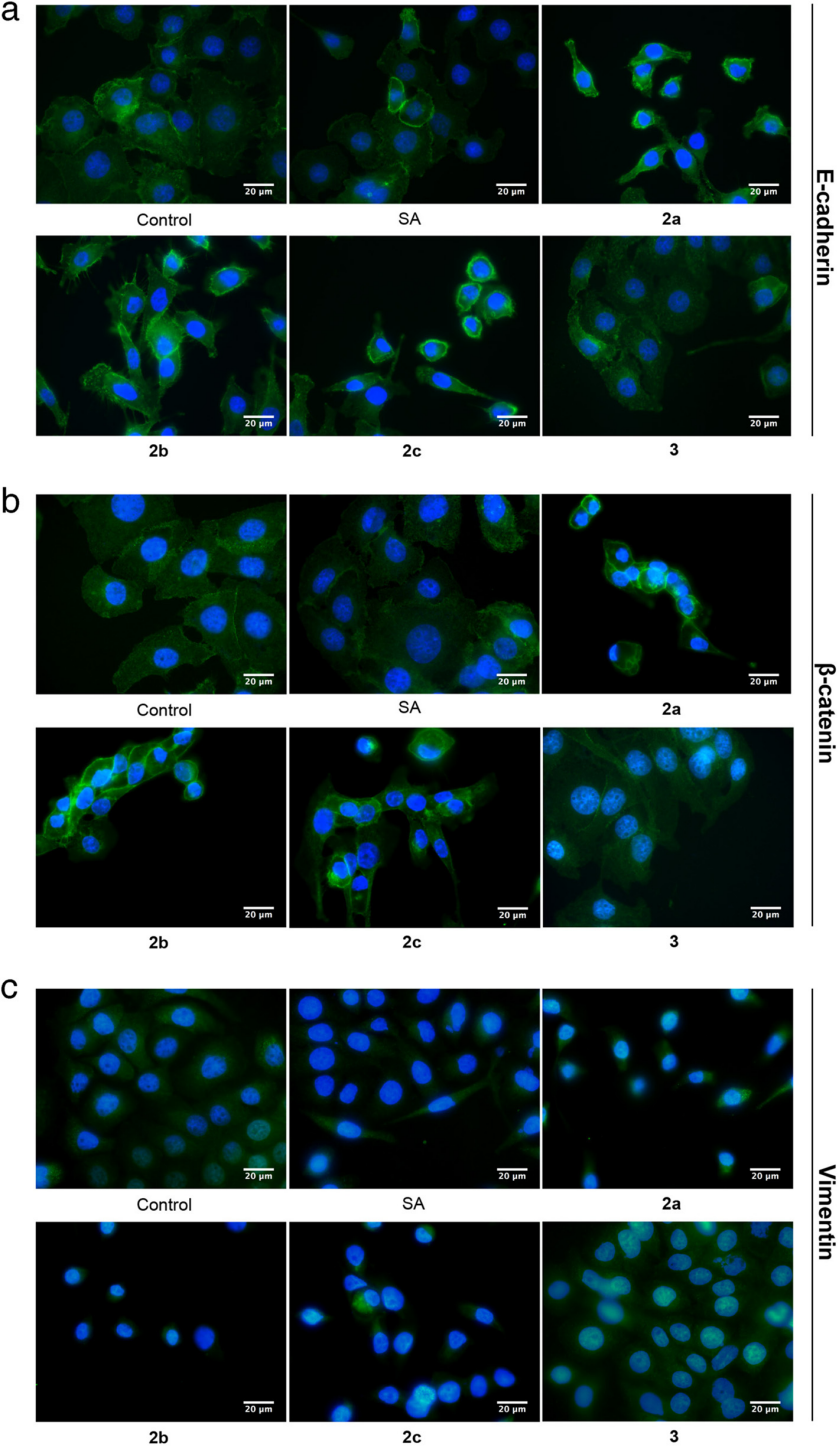
Treatment with the analogs **2a**–**c** induce expression of E-cadherin and β-catenin at the cell surface. The cells were treated with a 50 nM concentration of the compounds for 72 h. **a**–**c** E-cadherin, β-catenin and vimentin, respectively, detected with immunofluorescence microscopy after appropriate labeling with specific primary antibodies followed by secondary Alexa Fluor 488-conjugated antibodies (green). Nuclei were stained with bisbenzimide (blue). Bars = 20 μm. SA: salinomycin, **2a**: carbamate, **2b**: acetate, **2c**: carbonate and **3**: C1-methyl ester

Treatment with C20 analogs for 72 h increased the E-cadherin and β-catenin expression levels at the cell membrane while treatment with salinomycin and the C1-methyl ester did not give a noticeable effect on these protein levels (Fig. 3a and b). The expression of vimentin was decreased both by treatment with the C20 analogs and by salinomycin (Fig. 3c).

Taken together, the results show that the C20 analogs were more efficient at a 50 nM concentration than salinomycin in increasing the expression of the epithelial markers at the cell surface. All compounds tested except the C1-methyl ester also decreased the expression of the mesenchymal marker vimentin at this concentration.

### Treatment with C20-*O*-acylated analogs reduce cell migration

**Fig. 4:**
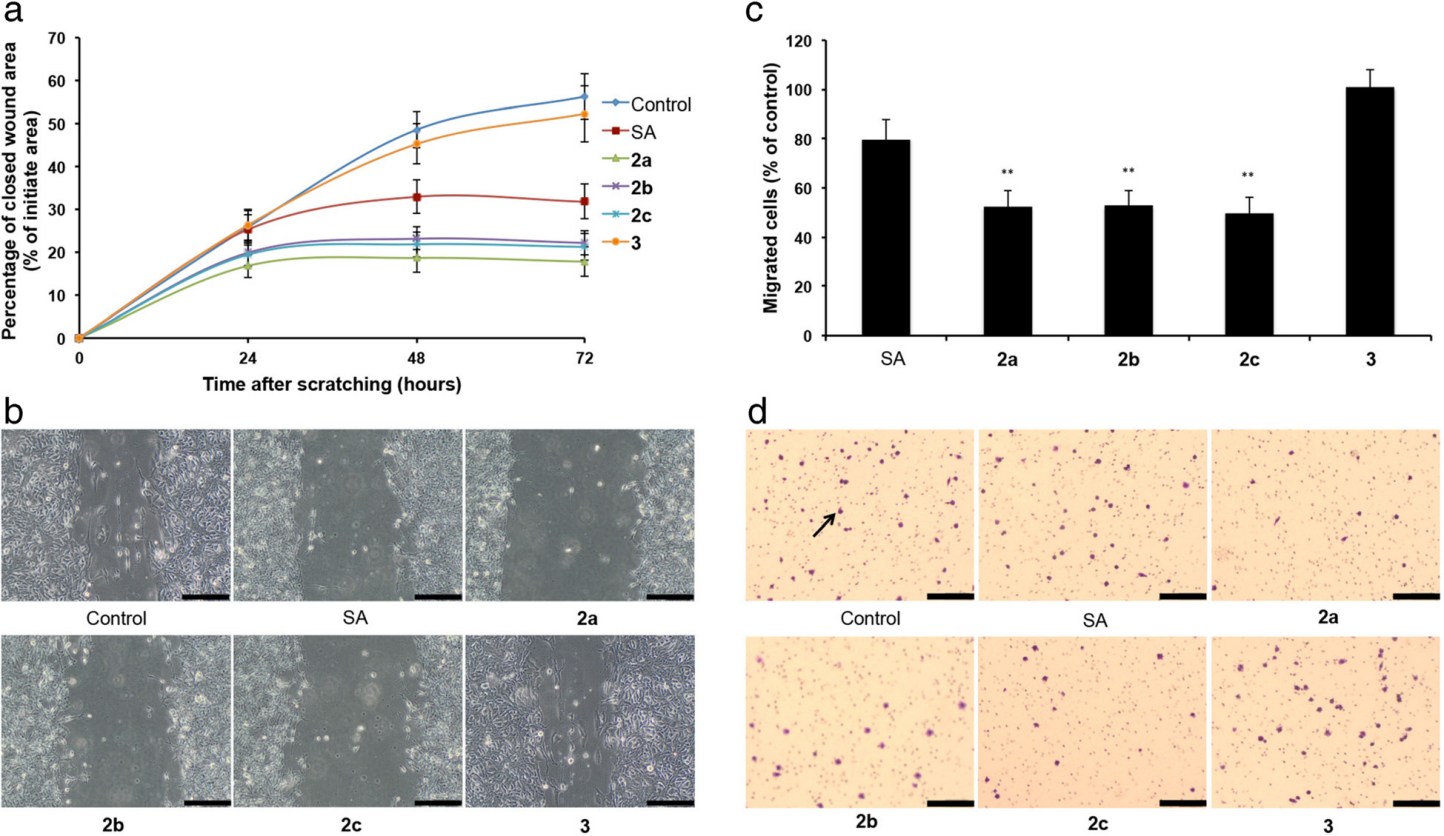
Cell migration after treatment with a 50 nM concentration of salinomycin or salinomycin analogs **2a**-**c. a** Quantification of wound healing. **b** Representative images of wound healing at 72 h after scratching. **c** Quantification of migrated cells in a Boyden chamber cell migration assay after 24 h of treatment. **d** Representative images of migrated cells. JIMT-1 cells were plated in trans wells with membranes containing 8.0 μm pores in medium containing the indicated compound. After 24 h of treatment, cells that had migrated to the bottom of the trans well were stained and photographed. The arrow points to a migrated cell. The small dots are the pores. Data are represented as mean ± SEM for *n* = 5. Bars in (**b**) and (**d**) = 200 μm. SA: salinomycin, **2a**: carbamate, **2b**: acetate, **2c**: carbonate and **3**: C1-methyl ester. **P < 0.01 compared to control

A decreased expression of vimentin and increased expression of E-cadherin and β-catenin at the cell surface should lead to a cellular functional effect manifested in decreased migration. We thus investigated the effect of both the C20 analogs and salinomycin on cell migration using a 50 nM concentration in wound-healing and Boyden chamber assays. Both assays showed that the acylated analogs as well as salinomycin reduced cell migration (Fig. 4). However, each of the acylated analogs was more efficient in inhibiting wound closure and cell migration than salinomycin at this concentration. The C1-methyl ester had no effect on migration compared to control.

### Salinomycin and the C20 analogs exert CSC and cell cycle effects through the same mechanism of action

**Fig. 5:**
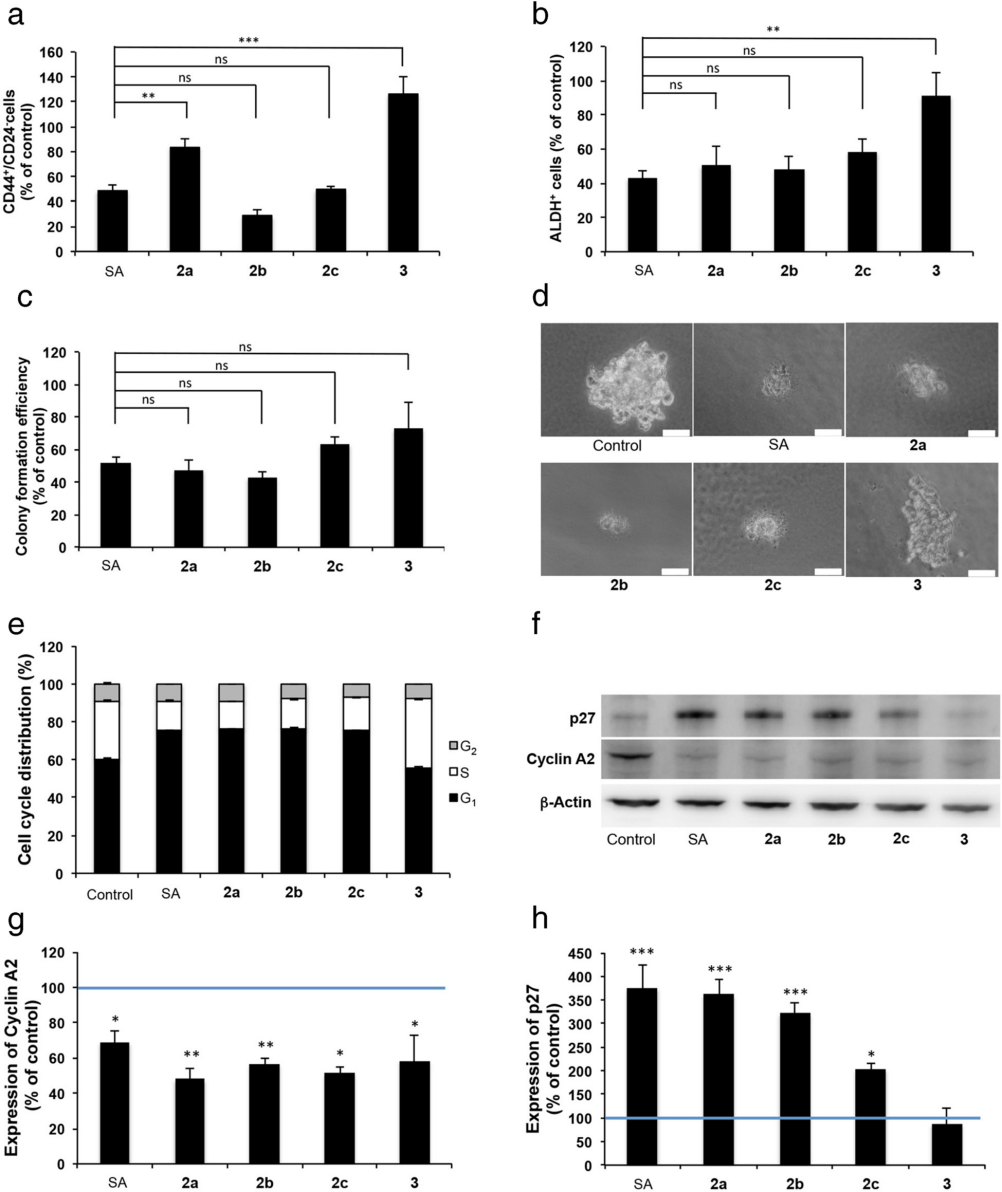
Treatment with IC_50_ of salinomycin and the analogs **2a**-**c** result in similar effects on CSCs and cell proliferation. **a** The CD44^+^/CD24^−^ population evaluated by flow cytometry after 72 h of treatment. **b** The ALDH^+^ population evaluated by flow cytometry after 72 h of treatment. **c** Colony forming efficiency evaluated using a serum free soft agar assay. The cells were treated for 72 h and then reseeded at cloning density. The colonies were counted after 2 weeks of incubation. **d** Colonies obtained from the serum free soft agar assay. Bars = 40 μm. **e** Cell cycle phase distribution evaluated by flow cytometry. **f** Representative Western blots used for densitometric scanning. **g**–**h** Expression of cyclin A2 and p27, respectively. Data are represented as mean ± SEM for n ≥ 3. The columns in (**a**–**d**) show mean ± SEM for *n* = 4. *P < 0.05, **P < 0.01, ***P < 0.001. ns: no significant difference. SA: salinomycin, **2a**: carbamate, **2b**: acetate, **2c**: carbonate and **3**: C1-methyl ester

Compounds that exert activity through the same molecular mechanism should give similar effects upon treatment at IC_50_. When treating cells with each of the analogs and salinomycin at the respective IC_50_ (Fig. 1), all compounds except the C1-methyl ester (used at a 20 μM concentration) reduced the CD44^+^/CD24^−^ population to a similar extent. Moreover, treatment at IC_50_ also decreased the ALDH^+^ cell population to around 50 % of control while the C1-methyl ester at a 20 μM concentration was inactive in this assay (Fig. 5a and b, Additional files 5 and 6: Figure S5 and S6). The same trend translated also to colony formation, although the C1-methyl ester at 20 μM gave a slight decrease in colony forming efficiency (Fig. 5c and d). The C1-methyl ester is a xenobiotic and is used at a high concentration compared to the other compounds and thus some toxicity is not surprising. When compared to control, treatment with all compounds including the C1-methyl ester resulted in a significant decrease in the number of colonies formed (*p* < 0.0001 for SA, **2a** and **2b**; *p* = 0.0002 for **2c**; *p* = 0.007 for **3**).

We also investigated the effects on cell cycle phase distribution and induction of cell death. When the cells were treated at the IC_50_ of salinomycin or the C20 analogs, the G_1_ phase increased and the S phase decreased (Fig. 5e). In contrast, cultures treated with the C1-methyl ester showed the same cell cycle phase distribution as control (Fig. 5e). None of the compounds induced cell death as evidenced by the absence of a sub-G_1_ signal in the DNA histogram (not shown). To gain insight into the molecular cause of the increase in the G_1_ phase, proteins that have major roles in cell cycle progression through the G_1_ and S phases were investigated by Western blot analysis. The levels of cyclins D1 and E1, important for G_1_ progression and G_1_/S transition, respectively, did not change compared to control after treatment with any of the compounds (not shown). However, the level of cyclin A2, important for S phase progression, was significantly reduced by all compounds (Fig. 5f and g). The decrease in cyclin A2 is thus a reflection of a decreased S phase. The G_1_ accumulation in cells treated with IC_50_ of salinomycin or the C20 acylated analogs can be attributed to the significantly increased expression of the CDK/cyclin inhibitor p27 (Fig. 5f and h). The p27 level was not affected in cells treated with the C1-methyl ester. Expression of the CDK/cyclin inhibitor p21 was not affected by treatment with any of the compounds (not shown).

The cell cycle investigation shows that treatment with a 50 nM concentration of the compounds resulted in a slight increase in the G_1_ phase of **2c**-treated cells compared to control while treatment with the other compounds resulted in a similar cell cycle phase distribution as control (Additional file 7: Figure S7). The level of cyclin A2 was the same in all treatment groups while p27 was increased in cells treated with **2b** and **2c** but substantially less compared to treatment with IC_50_ concentrations (compare Fig. 5 with Additional file 7: Figure S7). Analysis of the sub-G1 peak of DNA histograms did not show any sub-G1 peak as evidence of cell death in any of the treatment groups at a 50 nM concentration (not shown).

## Discussion

Using several independent CSC assays, a series of synthetic C20-*O*-acylated analogs of salinomycin reduced the putative CSC population in the JIMT-1 breast cancer cell line already at a 50 nM concentration where salinomycin itself does not show activity. The synthetic analogs are thus significantly more active against CSCs than the native structure. The mechanism of action appears to be the same for all these compounds as several independent assays show that salinomycin and the C20 analogs give similar responses at the respective IC_50_.

Although salinomycin has been investigated extensively in a CSC context in recent years, the exact mechanism of action of this compound is not well understood. To this end, it is important that the C1-methyl ester analog of salinomycin, a compound that retains the molecular structure of salinomycin, but displays an over three orders of magnitude reduced ion-binding ability [31] and is essentially incapable of charge-neutral ion transport, does not influence CSC-related properties even when used at higher concentrations. This gives experimental credence to the molecular initiating event for salinomycin and its C20 analogs as being related to disturbed membrane dependent ion gradients. These changes presumably then influence various signal transduction pathways and cell functions, which subsequently result in a decrease in the number of CSCs as well the mesenchymal traits of the cell population. Our data thus emphasize a significant role of the antiporter capacity of salinomycin and its more active C20 analogs rather than interaction with a specific cellular target. This notion is further corroborated by the prior observations that the related ionophores monensin and nigericin have similar effects as salinomycin [7, 52]. The nature of the properties required for the high activity against CSCs of such structures however remains an open question. In particular, a potential connection between the CSC response and the alkali ion binding selectivity and transport efficiency is of interest in this context.

In addition to self-renewal and differentiation, CSCs have been shown to possess increased metastatic capacity, which is related to the process of EMT [53]. Salinomycin treatment has previously been shown to inhibit the ability of several cancer cell lines including prostate, colorectal and human bone marrow-derived mesenchymal stem cells to migrate when used at concentrations ranging from 50 nM to 10 μM [9, 11, 14, 22, 54, 55]. Our results show that the C20 analogs inhibited cell migration even more efficiently than salinomycin at a 50 nM concentration. Additionally, the expression of the epithelial markers E-cadherin and β-catenin at the cell surface was increased and the expression of the mesenchymal marker vimentin was decreased after treatment with the C20 analogs **2a**-**c**. In contrast, treatment with 50 nM salinomycin did not affect the expression of E-cadherin and β-catenin at the cell surface. The vimentin levels decreased after salinomycin treatment, which may explain the decreased rate of migration shown in the wound-healing and Boyden chamber assays although this effect was less pronounced than for the C20 analogs. Our data thus show that treatment with a 50 nM concentration of the analogs **2a**-**c** increased the number of cells with epithelial phenotype while cells with mesenchymal phenotype decreased. In principle, the origin of this effect may be an induction of mesenchymal to epithelial transition (MET) i.e. a change in phenotype, but the same outcome would be achieved if there were selective death or growth inhibition of mesenchymal cells. An investigation of the sub-G_1_ region in the cell cycle phase distribution of DNA histograms did not show induction of cell death upon treatment with a 50 nM concentration of the analogs. As cell proliferation is slightly inhibited at IC_25_, the results can be interpreted as originating from inhibition of proliferation of mesenchymal cells and induction of MET in these cells. Similar results have been obtained when treating JIMT-1 cells with the anticancer polyamine analog PG11047 [42].

More active salinomycin analogs are of interest in light of the recent case reports where salinomycin treatment resulted in partial tumor and metastasis regression of breast cancer [28, 29]. Our results show that the synthetic C20 analogs exhibited improved activity against breast CSCs compared to salinomycin using several well-accepted traits of CSCs. These compounds moreover efficiently induced MET resulting in a decreased capacity of cell migration, a property closely related with metastasis of cancer. Importantly, these effects were seen for the synthetic C20 analogs already at low nanomolar concentrations where salinomycin itself was inactive. In particular, acetate **2b** and the hydrolytically more stable ethyl carbonate **2c** are attractive for further investigations as these compounds display similar enhanced activities in both MTT and CSC assays and can moreover be readily synthesized in high yields from abundantly available salinomycin [30].

## Conclusions

We have previously shown that salinomycin derivatives, readily available in just a few synthetic steps, are significantly more active than salinomycin against breast cancer cells. Herein we show that these improvements in activity also translate to an enhanced selectivity against CSCs already at low nanomolar concentrations where salinomycin itself is inactive as shown by complementary marker-based and functional cell assays. Additionally, we show that traits associated with mesenchymal cells including cell migration and vimentin expression are efficiently reduced by the analog structures at low concentrations, while epithelial traits such as E-cadherin and β-catenin expression at the cell surface are increased reflecting a mesenchymal to epithelial transition. The similar responses across the assays when treating cells at the respective IC_50_ strengthens that salinomycin and its C20-acylated analogs, although varying in potency, exert their influence on the CSC population through a shared mechanism. While a number of downstream effects have been invoked in explaining the activity of salinomycin against CSCs, the molecular initiating event has remained unclear. Using the synthetic structural analogs as mechanistic probes, we lend experimental credence to ionophoric stress as the origin of the observed inhibition of CSCs. We anticipate that structural analogs of salinomycin that elicit similar cellular responses compared to the native structure but at significantly lower concentrations will be of immediate value both towards clinical relevance and for further mechanistic and biological investigations.

## Additional files

Additional file 1: Figure S1. Representative cytograms of cell surface markers CD44 and CD24 obtained using flow cytometry. JIMT-1 cells were treated with 50 nM salinomycin or salinomycin analogs for 72 h. SA: salinomycin, 2a: carbamate, 2b: acetate, 2c: carbonate and 3: C1-methyl ester. (DOCX 1340 kb) Additional file 2: Figure S2. U-shaped dose response curve found for the CD44^+^ /CD24^−^ population in *N*–ethyl carbamate 2a-treated JIMT-1 cells. JIMT-1 cells were treated with 2a for 72 h at the indicated concentrations. The effect on the CD44^+^/CD24^−^ population was determined using flow cytometry. Data are represented as mean ± SEM for *n* = 4. (DOCX 41 kb) Additional file 3: Figure S3. Representative cytograms of ALDH assay obtained using flow cytometry. JIMT-1 cells were treated with 50 nM salinomycin or salinomycin analogs for 72 h. SA: salinomycin, 2a: carbamate, 2b: acetate, 2c: carbonate and 3: C1-methyl ester. (DOCX 1678 kb) Additional file 4: Figure S4. Salinomycin treatment decreases the proportion of ALDH^+^ in a dose dependent manner. JIMT-1 cells were treated with salinomycin for 72 h at the indicated concentrations. The effect on the ALDH^+^ population was determined using flow cytometry. Data are represented as mean ± SEM for *n* = 3. (DOCX 38 kb) Additional file 5: Figure S5. Representative cytograms of cell surface markers CD44 and CD24 obtained using flow cytometry. JIMT-1 cells were treated with salinomycin or salinomycin analogs at IC_50_ for 72 h. SA: salinomycin, 2a: carbamate, 2b: acetate, 2c: carbonate and 3: C1-methyl ester. (DOCX 1331 kb) Additional file 6: Figure S6. Representative cytograms of ALDH assay obtained using flow cytometry. JIMT-1 cells were treated with salinomycin or salinomycin analogs at IC_50_ for 72 h. SA: salinomycin, 2a: carbamate, 2b: acetate, 2c: carbonate and 3: C1-methyl ester. (DOCX 1597 kb) Additional file 7: Figure S7. Cell cycle effects of treating with 50 nM salinomycin or the analogs 2a-c for 72 h. (a) Cell cycle phase distribution evaluated using flow cytometry. (b) Representative Western blots used for densitometric scanning to obtain the data in (c) and (d). (c-d) Expression of cyclin A2 and p27, respectively. The columns in (c) and (d) show mean ± SEM for *n* = 6. * *P* < 0.05. SA: salinomycin, 2a: carbamate, 2b: acetate, 2c: carbonate and 3: C1-methyl ester. (JPG 737 kb)

## Abbreviations

ALDH: aldehyde dehydrogenase
CSCs: cancer stem cells
DEAB: diethylaminobenzaldehyde
DMSO: dimethyl sulfoxide
EMT: epithelial to mesenchymal transition
MET: mesenchymal to epithelial transition
PBS: phosphate-buffered saline
SA: salinomycin
