# Supplementary material for: Breast cancer stem cell selectivity of synthetic nanomolar-active salinomycin analogs
Source: BMC Cancer. 2016 Feb 23;16:145. doi: 10.1186/s12885-016-2142-3 (PMC4765157; doi:10.1186/s12885-016-2142-3)
Supplement: Additional file 2: Figure S2. — U-shaped dose response curve found for the CD44+ /CD24− population in N–ethyl carbamate 2a-treated JIMT-1 cells. JIMT-1 cells were treated with 2a for 72 h at the indicated concentrations. The effect on the CD44+/CD24− population was determined using flow cytometry. Data are represented as mean ± SEM for n = 4. (DOCX 41 kb) [file 12885_2016_2142_MOESM2_ESM.docx]

**Figure S2.** U-shaped dose response curve found for the CD44^+^/CD24^-^ population in N–ethyl carbamate **2a**-treated JIMT-1 cells. JIMT-1 cells were treated with **2a** for 72 h at the indicated concentrations. The effect on the CD44^+^/CD24^-^ population was determined using flow cytometry. Data are represented as mean ± SEM for n = 4.
