# Supplementary material for: Breast cancer stem cell selectivity of synthetic nanomolar-active salinomycin analogs
Source: BMC Cancer. 2016 Feb 23;16:145. doi: 10.1186/s12885-016-2142-3 (PMC4765157; doi:10.1186/s12885-016-2142-3)
Supplement: Additional file 4: Figure S4. — Salinomycin treatment decreases the proportion of ALDH+ in a dose dependent manner. JIMT-1 cells were treated with salinomycin for 72 h at the indicated concentrations. The effect on the ALDH+ population was determined using flow cytometry. Data are represented as mean ± SEM for n = 3. (DOCX 38 kb) [file 12885_2016_2142_MOESM4_ESM.docx]

**Figure S4.** Salinomycin treatment decreases the proportion of ALDH^+^ in a dose dependent manner. JIMT-1 cells were treated with salinomycin for 72 h at the indicated concentrations. The effect on the ALDH^+^ population was determined using flow cytometry. Data are represented as mean ± SEM for n = 3.
