# Supplementary material for: Breast cancer stem cell selectivity of synthetic nanomolar-active salinomycin analogs
Source: BMC Cancer. 2016 Feb 23;16:145. doi: 10.1186/s12885-016-2142-3 (PMC4765157; doi:10.1186/s12885-016-2142-3)
Supplement: Additional file 6: Figure S6. — Representative cytograms of ALDH assay obtained using flow cytometry. JIMT-1 cells were treated with salinomycin or salinomycin analogs at IC50 for 72 h. SA: salinomycin, 2a: carbamate, 2b: acetate, 2c: carbonate and 3: C1-methyl ester. (DOCX 1597 kb) [file 12885_2016_2142_MOESM6_ESM.docx]

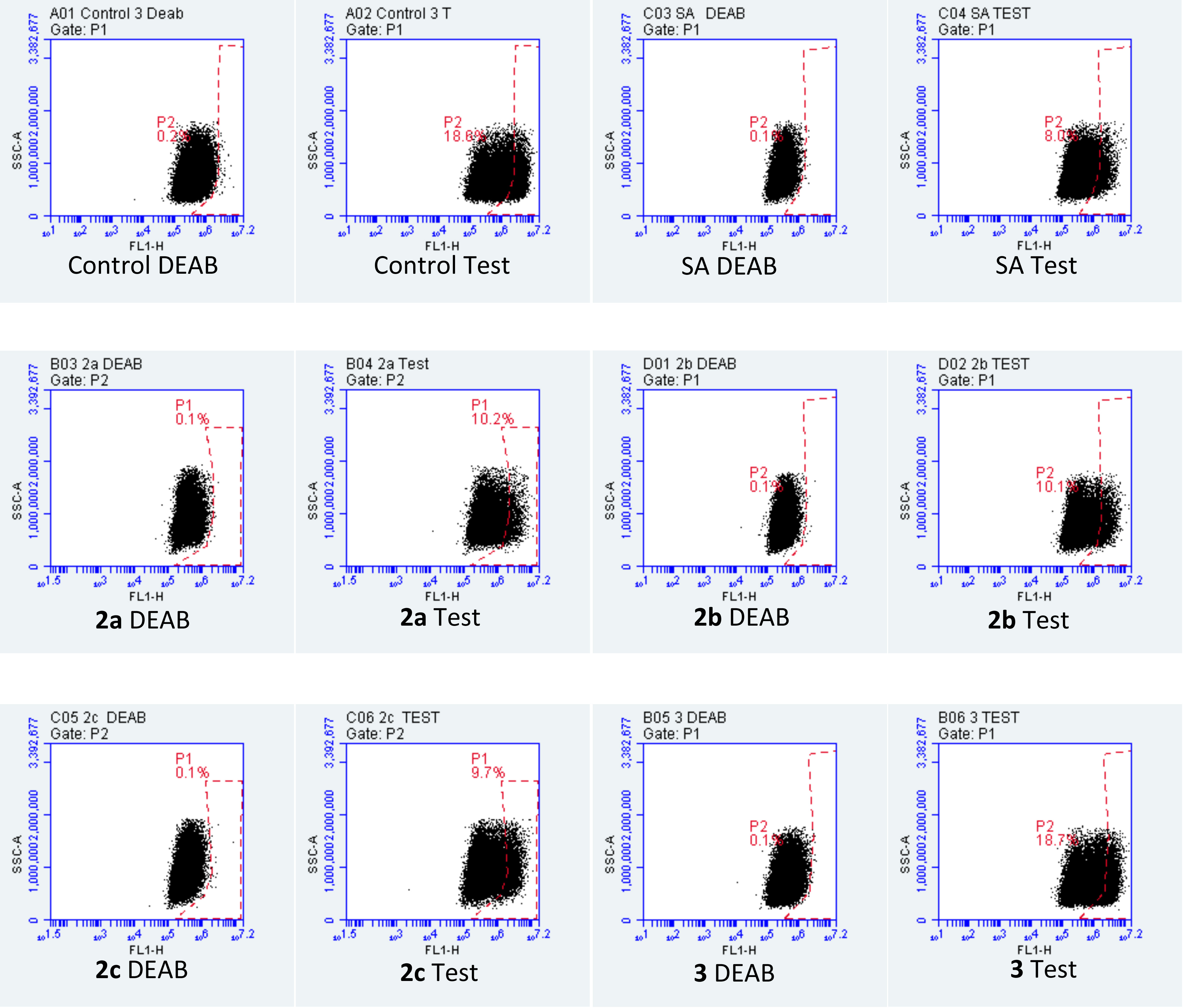


**Figure S6.** Representative cytograms of the ALDEFLUOR assay obtained using flow cytometry. JIMT-1 cells were treated with salinomycin or salinomycin analogs at IC_50_ for 72 hours. SA: salinomycin, **2a**: carbamate, **2b**: acetate, **2c**: carbonate, and **3**: C1-methyl ester.
