# Supplementary figures and images for: Breast cancer stem cell selectivity of synthetic nanomolar-active salinomycin analogs
Source: BMC Cancer. 2016 Feb 23;16:145. doi: 10.1186/s12885-016-2142-3 (PMC4765157; doi:10.1186/s12885-016-2142-3)

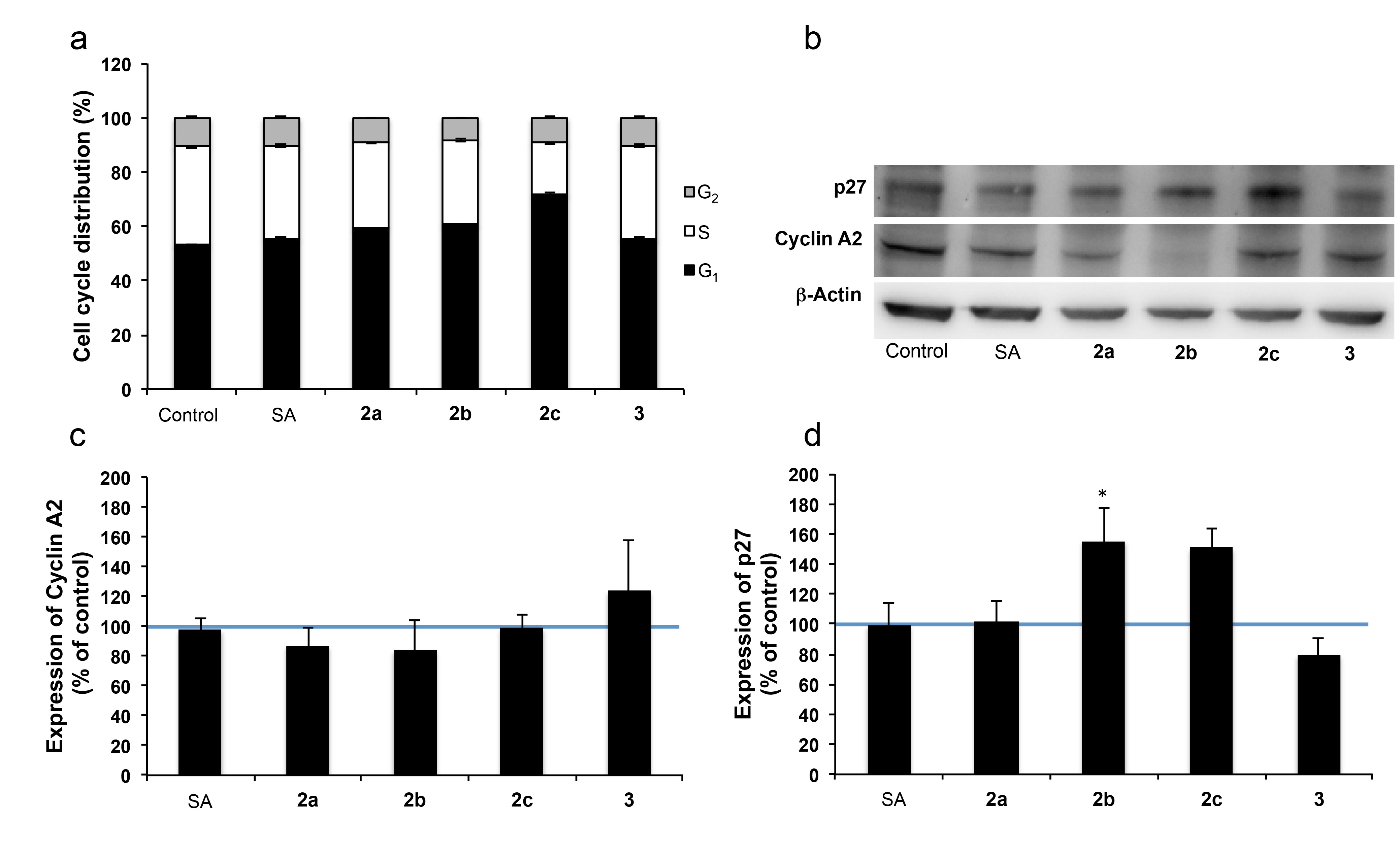

Supplement: Additional file 7: Figure S7. — Cell cycle effects of treating with 50 nM salinomycin or the analogs 2a-c for 72 h. (a) Cell cycle phase distribution evaluated using flow cytometry. (b) Representative Western blots used for densitometric scanning to obtain the data in (c) and (d). (c-d) Expression of cyclin A2 and p27, respectively. The columns in (c) and (d) show mean ± SEM for n = 6. * P < 0.05. SA: salinomycin, 2a: carbamate, 2b: acetate, 2c: carbonate and 3: C1-methyl ester. (JPG 737 kb) [file 12885_2016_2142_MOESM7_ESM.jpg]
